# Clinical Outcomes of Root-Analogue Implants Restored with Single Crowns or Fixed Dental Prostheses: A Retrospective Case Series

**DOI:** 10.3390/jcm9082346

**Published:** 2020-07-23

**Authors:** Mats Wernfried Heinrich Böse, Detlef Hildebrand, Florian Beuer, Christian Wesemann, Paul Schwerdtner, Stefano Pieralli, Benedikt Christopher Spies

**Affiliations:** 1 Department of Prosthodontics, Geriatric Dentistry and Craniomandibular Disorders, Institute for Dentistry, Oral and Maxillofacial Surgery, Charité—Universitätsmedizin Berlin, Corporate Member of Freie Universität Berlin, Humboldt-Universität zu Berlin, and Berlin Institute of Health, Campus Benjamin Franklin (CBF), Aßmannshauser Str. 4–6, 14197 Berlin, Germany; florian.beuer@charite.de (F.B.); christian.wesemann@charite.de (C.W.); 2 Private Dental Office Dr. Detlef Hildebrand, Westhafenstraße 1, 13353 Berlin, Germany; hildebrand@dentalforum-berlin.de; 3 Institute of Mathematics MA 4–5, Technical University Berlin, Straße des 17. Juni 136, 10623 Berlin, Germany; schwerdt@math.tu-berlin.de; 4 Department of Prosthetic Dentistry, Center for Dental Medicine, Medical Center—University of Freiburg, Faculty of Medicine—University of Freiburg, Hugstetter Str. 55, 79106 Freiburg, Germany; stefano.pieralli@uniklinik-freiburg.de (S.P.); benedikt.spies@uniklinik-freiburg.de (B.C.S.)

**Keywords:** root-analogue implants, customized implants, CAD/CAM, dental implants, prosthodontics

## Abstract

The objective was to investigate clinical and radiological outcomes of rehabilitations with root-analogue implants (RAIs). Patients restored with RAIs, supporting single crowns or fixed dental prostheses, were recruited for follow-up examinations. Besides clinical and esthetical evaluations, X-rays were taken and compared with the records. Patients were asked to evaluate the treatment using Visual Analogue Scales (VAS). For statistical analyses, mixed linear models were used. A total of 107 RAIs were installed in one dental office. Of these, 31 were available for follow-up examinations. For those remaining, survival has been verified via phone. RAIs were loaded after a mean healing time of 6.6 ± 2.5 months. 12.1 ± 6.9 months after loading, a mean marginal bone loss (MBL) of 1.20 ± 0.73 mm was measured. Progression of MBL significantly decreased after loading (*p* = 0.013). The mean pink and white esthetic score (PES/WES) was 15.35 ± 2.33 at follow-up. A survival rate of 94.4% was calculated after a mean follow-up of 18.9 ± 2.4 months after surgery. Immediate installation of RAIs does not seem to reduce MBL, as known from the literature regarding screw-type implants, and might not be recommended for daily routine. Nevertheless, they deliver esthetically satisfying results.

## 1. Introduction

Dental implants are successfully used for the replacement of missing and hopeless teeth with a mean survival rate of 94.6 ± 5.97% after 10 years of clinical service [1]. Concerning the loss of adjacent bone and soft tissues as a result of tooth extraction [2], immediate implant placement has been purported to help reducing resorption processes [3]. However, a generalized recommendation whether immediate, immediate-delayed, or delayed installation of prefabricated screw-shaped implants should be generally preferred was not scientifically evidenced to date [4,5]. Benefits of immediate surgical procedures are still subject to current investigations [6].

Resorption processes after tooth extraction have been part of scientific research since the twentieth century [7]. With the objective to counteract these processes by replacing extracted roots/teeth with anatomically shaped copies, the Dental Polymer Implant Concept was already introduced in 1969 [8]. Since then, scientific research on root-analogue implants (RAIs) have been conducted repeatedly with variations in used materials and methods in the literature [9,10,11,12,13,14,15,16,17]. Thereby, the technical possibilities and concepts did indeed allow for the fabrication of root-analogues but not for immediate implant installation in the proper meaning of the word. This resulted in a temporal offset between the removal of the teeth and implant installations. In order to allow for immediate implant installation of RAIs, they needed to be manufactured prior to the extractions. With the help of Digital Imaging and Communication in Medicine (DICOM) data gathered from Cone Beam Computed Tomographies (CBCT), accurate production of RAIs has been made possible prior to surgery [18,19]. However, no long-term clinical data and studies are available, allowing for critical assumptions regarding the former treatment concepts.

In 2013, Natural Dental Implants (NDI Berlin) launched the REPLICATE Immediate Tooth Replacement System on the dental market. Based on the combination of DICOM data and Standard Triangulation/Tesselation Language (STL) data of the intraoral clinical situations, the RAIs were manufactured in a subtractive manner prior to extraction/surgery. Regarding material selection, a one-piece hybrid-version (the endosseous part of the implant is made of titanium and glass-fused to the zirconia abutment) and a one-piece zirconia version was available. The entire endosseous implant surface was enlarged by macro and micro retentions to allow for primary stability during re-installation of the replica. In 2017, a case report described an excellent outcome concerning the clinical and radiographic results [20]. Some authors compared the procedure to the so-called “The Digital One-Abutment/One-Time Concept” [21], another approach aiming to preserve soft and hard tissues using screw-shaped implants. As a result, more clinical data regarding the REPLICATE Immediate Tooth Replacement System was deemed necessary in 2018 [22]. This investigation intended to provide more clinical data and form the basis for further prospective studies on RAIs. The collected data is considered valuable for future investigations and forthcoming developments of root-analogue implant systems. Since the concept of RAIs fulfils the claim for customization and the belief in progressive digitization within dentistry [23], upcoming research on fully individualized implants seems likely.

This retrospective case series was designed to investigate the survival and success rates of rehabilitations with the REPLICATE Immediate Tooth Replacement System seeking for potential clinical, radiological, and social impacts. With a focus on marginal bone loss (MBL) and esthetic outcomes, the objective was to identify advantages and/or disadvantages and provide preliminary data to validate routine clinical applicability. The working hypothesis was that immediate implantations with customized root-analogue implants show comparable clinical, radiographical, and esthetical results to conventional screw-shaped implants.

## 2. Experimental Section

This study was designed as a clinical retrospective case series. Ethical approval was given by the Ethical Committee of Charité—Universitätsmedizin Berlin, Germany (application number: EA4/140/18). All surgical procedures and follow-up examinations were performed in the private dental office of an experienced dentist focusing on oral surgery in Berlin (author D.H.). D.H. performed all surgical procedures, and the author, M.B., performed all clinical follow-up examinations. All patients received and signed an informed consent form and patient information prior to examinations. In addition to the Case Report Forms (CRFs) completed by the investigator, each participant was asked to assess his own perception of the treatment by applying Visual Analogue Scales (VAS). This research was conducted considering the STROBE statement for observational studies.

To be considered for a treatment with a RAI in the dental office of the study, the patients had to be non-smokers and were not allowed to take any medication affecting the bone metabolism. Regarding the unmaintainable teeth, surrounding soft and hard tissues had to be non-inflammatory, surrounding bone compartments had to be intact, and periodontal gaps had to be visible on CBCTs. The surgeon (D.H.) already conducted the selection of patients during implant consultations, and many RAI surgeries were already performed before the development of the present study. Rejected cases for a RAI treatment were not documented in the dental office. In total, 107 RAIs were inserted between September 2016 and August 2019 in the dental office of the study. Treatments were performed on patients, with an agreement to be treated with a RAI, and met the inclusion criteria. They were referred by their general dentists from all over Germany or introduced themselves on their own initiative. The REPLICATE Immediate Tooth Replacement System had already been approved for the German dental market at the time of all surgeries.

**Figure 1:**
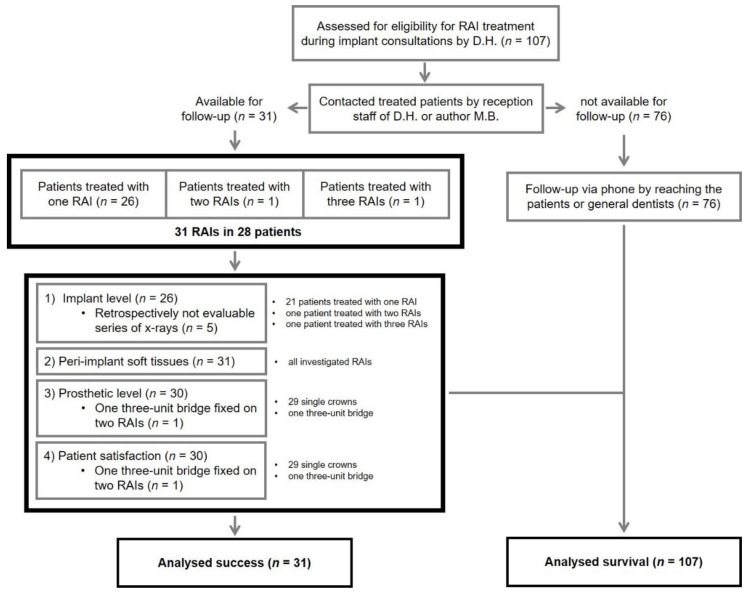
Flow diagram regarding study design and distribution; *n*: number of root-analogues/evaluated data within the different stages of the present study.

Existing and collected data were anonymized, retrospectively analyzed, and statistically evaluated comparing the patients CRFs and VAS ratings with the detailed records deemed necessary from the manufacturer. Patient inclusion criteria for clinical follow-up examinations in this study were: (1) Treated with the REPLICATE Immediate Tooth Replacement System; (2) completed healing after surgery according to the surgeon; and (3) RAIs restored with single crowns or fixed dental prostheses. All patients treated with a RAI in the dental office refusing to join for follow-up were excluded from the statistical analyses. However, they were included in the calculation of a preliminary overall survival rate in case of confirmation of survival by the patients themselves or the records of the referring dentists. All patients were tried to contact by phone repeatedly by the reception staff of D. H. or the author, M. B. However, only 28 patients treated with 31 RAIs agreed and participated in the follow-up investigations. The reduced study participation compared to total surgeries emphasizes the study practice as part of a dental referral network and the widely dispersed residencies of the treated patients. The dental office was one of a few practices that offered a treatment with this system throughout Germany. Figure 1 provides an overview of this study as a flow diagram.

Detailed planning was required prior to surgical procedures. To fabricate the RAIs, dental impressions with a customized tray and a polyether material (Impregum, 3M Deutschland GmbH, Neuss, Germany), a bite record in habitual occlusion (Futar D, Kettenbach GmbH & Co. KG, Eschenburg, Germany), DICOM data from a CBCT (PAX i-3D, VATECH, Hwaseong-si, Gyeonggi-do, South Korea), completed order forms, and clinical photographs were required by NDI Berlin. Models were fabricated (type IV plaster), digitized with a laboratory scanner, and exported in STL data format. The STL and DICOM data were superimposed by a trained specialist of the manufacturer. Temporary Protective Covers (TPCs), implant portions, abutment portions, and Try-Ins (exact copies of the RAIs to evaluate the fit prior to installation) were virtually designed. Both the design engineer and the dentist checked the design of the datasets. Depending on the dentist’s preferences, adjustments to the design were carried out. When approved, the DICOM data were converted into STL datasets and sent to the Computer Aided Manufacturing (CAM)-computers, which generated the Network Common Data Format (NetCDF; nc) to control the milling machines. Regardless of hybrid or all-ceramic RAIs, the surfaces of the implant portions were enlarged using macro and micro retentions. To avoid compression of the alveolar bone, the intra-bony part was individually reduced compared to the original size of the root.

**Figure 2:**
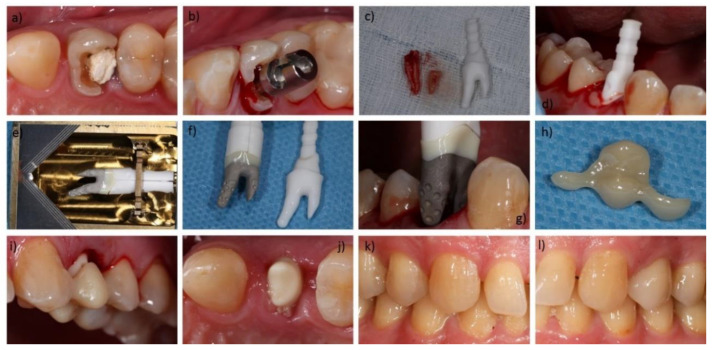
Exemplary workflow of a restoration with the *REPLICATE Immediate Tooth Replacement System*: (**a**) Tooth (24/UL4) not maintainable with longitudinal fracture; (**b**) Inserted Benex Extraction-System (Benex Extraction-System, Helmut Zepf Medizintechnik GmbH, Seitlingen-Oberflacht, Germany); (**c**) extracted root and Try-In; (**d**) Try-In in the alveolus with Plasma Rich Growth Factors (PRGF) at the buccal junction; (**e**) root-analogue implant (RAI) in its opened sterile packaging; (**f**) RAI with placement assistance compared to the Try-In; (**g**) installation of the RAI; (**h**) Temporary Protective Covers (TPC) before bonding; (**i**) bonded TPC as load protection; (**j**) healed RAI with interfering gingiva; (**k**) contralateral natural teeth; (**l**) restored RAI and adjacent teeth.

After pre-surgical planning and delivery of the RAIs, immediate installation took place. Hopeless teeth (Figure 2a) were removed as atraumatically as possible to maintain the surrounding bone. Where possible, the Benex Extraction-System (Benex Extraction-System, Helmut Zepf Medizintechnik GmbH, Seitlingen-Oberflacht, Germany) was used (Figure 2b), facilitating atraumatic vertical tooth removal with a reported mean success rate of 83% [24]. If planned and necessary, the bone compartments were adjusted on an individual basis. Before unpacking and insertion of the final RAI, the anticipated fit in the extraction socket was evaluated by using the Try-Ins (Figure 2c,d). All implant surfaces were wetted with Plasma Rich Growth Factors (PRGFs; BTI Biotechnology Institute, San Antonio, Spain). Since PRGFs might promote bone regeneration, it was used for augmentation of the voids [25]. Thereafter, the RAIs were carefully placed into position with a hammer and a mallet (Figure 2e–g). Subsequent to surgery, supplied TPCs were adhesively attached to one or both adjacent teeth, depending on the design (Figure 2h,i). A gap of approximately 0.6 mm between the TPCs and the abutments of the RAIs served as load protection. To investigate the state of healing, examinations usually took place 3–6 month after surgery, whereas the temporaries were carefully removed, and X-rays were taken. After successful osseointegration (Figure 2j), conventional impressions of the abutment for the manufacturing of the final restorations were made. The prosthetic restorations of the RAIs were delivered in the study center and in the referring dental offices (Figure 2l).

When setting up this retrospective evaluation, follow-up examinations were scheduled as soon as possible in consultation with the patients. The CRFs included an update of the anamnesis, surgical, prosthetic, and implant-specific parameters. Radiographs and clinical photographs of the restorations were taken at the follow-up examinations. VAS’ were used for the assessment of patient-reported outcomes. Referring to a systematic review published in 2012, success was determined including 4 superordinate categories: (1) Implant level; (2) Peri-implant soft tissue; (3) Prosthetic level; (4) and Patient satisfaction; this considers the complexity of rehabilitations with implant supported restorations [26].

**Figure 3:**
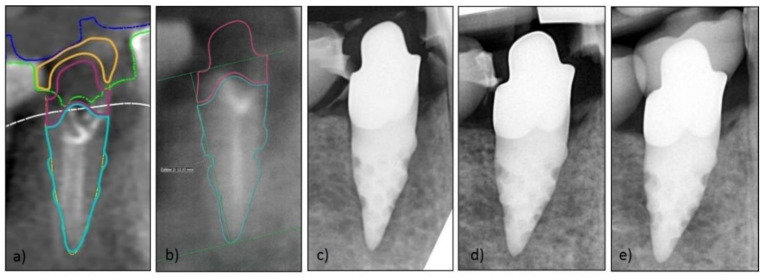
Exemplary series of X-rays for marginal bone loss (MBL) measurement: (**a**) visualized RAI construction by Natural Dental Implants (NDI); (**b**) initial image for measurement of bone loss; (**c**) X-ray post-implantation; (**d**) X-ray after healing time (before impression for loading); (**e**) X-ray after loading.

To evaluate success at the implant level radiographs were evaluated at three different times: (1) Surgery (T0); (2) (prior to) Loading (T1); and (3) Follow-up examination (T2). All relevant radiographs were analyzed with ImageJ, an open source image processing program designed for scientific multidimensional images (developed by Wayne Rasband). To calculate the bone loss, defined reference points of the digital RAI constructions (provided by NDI Berlin) were transferred to the radiographs and converted using parallels and the rule of three (Figure 3a–e). All points were defined and checked by the surgeon (D.H.) and the investigator (M.B.). They were independently measured by a single external private lecturer with experience in clinical studies to reduce subjective bias. Considering the measured bone loss, criteria according to Albrektsson et al. [27] and success criteria on the implant level (pain, bone loss < 1.5 mm at first year, annual bone loss < 0.2 mm thereafter, radiolucency, mobility, infection) [26] were used to evaluate clinical evidence of successful osseointegration. Furthermore the following parameters were investigated regarding their impact on bone loss: (1) Gender (male vs female); (2) Age; (3) Implant region (anterior vs posterior); (4) Implant location (maxilla vs mandible); (5) Implant material (hybrid vs all-ceramics); (6) Length of the one-piece implant; (7) Length of the root portion; (8) Length of the abutment portion; (9) Size of implant surface area; (10) Bone quality documented by the surgeon [28]; (11) Difficulty of the operation according to the surgeon (grouped into: easy, intermediate and complicated); and (12) number of roots.

To assess peri-implant soft tissues, the modified plaque (mPI) and bleeding indices (mBI) according to Mombelli et al. [29] and the width of keratinized gingiva (KG) were adopted as criteria at follow-up examinations. Both for mPI (scale 0–3) and mBI (scale 0–3), 0 and 1 were counted as success, whilst 2 and 3 were considered not successful. The keratinized gingiva had to maintain a width of at least 1.5 mm [26] for a rating as success.

The Pink and White Esthetic Score (PES/WES) [30] and the modified United States Public Health Service (USPHS) criteria (Table 1) [31] were used to allow for standardized assessment of the esthetic outcome and a success rating at the prosthetic level. For both PES and WES, the threshold for clinical acceptability was set at 6 [30] and regarded successful in this study. The 7 modified USPHS criteria: (1) Fracture of veneering ceramic; (2) Fracture of framework; (3) Occlusal roughness; (4) Marginal integrity; (5) Contour of reconstruction; (6) Esthetics of reconstruction; and (7) Discoloration of reconstruction, were applied to every reconstruction and each rated as Alpha (A: within a range of excellence), Beta (B: minor deviations from the ideal), Charlie (C: clinically unacceptable defects that could be intraorally repaired to a clinically acceptable level), or Delta (D: irreparable problem of clinical relevance).

Patient-reported outcomes were assessed by applying VAS. Assessment of appearance and chewing ability was included in the evaluation. The patients were asked to label a point on a line that corresponded with their personal satisfaction. The line was 10 cm in length, without scale, and every millimeter corresponded to 1% of satisfaction (i.e., 10 cm corresponded to 100%). The left endpoint represented poor satisfaction (0%), whereas the point at the right end represented excellent satisfaction (100%). Finally, the patient’s markings were measured with a ruler. A rating of 80% or more resulted in a rating as success.

For a successful RAI, all of the 4 superordinate criteria: (1) Implant level; (2) Peri-implant soft tissues; (3) Prosthetic level; and (4) Patient satisfaction, had to be classified successful themselves, following the above defined criteria.

Surgeries of a single practitioner (D.H.) were evaluated. In order to reduce bias, follow-ups were not conducted by the surgeon, but by another independent practitioner (M.B.). In addition, MBL measurements were carried out by an independent private lecturer with experience in clinical studies. The data collection was therefore performed preferably independently and without financial support from the implant company. When compiling the examination parameters, care was taken to use parameters that were as objective and scientifically accepted as possible. Due to the small indication group for a treatment with a RAI, the objective to provide preliminary data and the scarcity of documented data sets in the literature, all possible data sets were evaluated, despite their heterogeneity.

Using 2-sided 95% confidence intervals, a total sample size of 31 RAIs was investigated. For the key clinical data (surgical and RAI-specific parameters, peri-implant soft tissue parameters, MBL, PES, WES, PES/WES), the means, minima, maxima, and standard deviations were calculated. The focus of statistical analysis was the progression of bone loss over time and parameters that may have an impact on it. To compensate for heterogeneous measurement intervals, the mean gradient of the bone loss during time interval 1 (from surgery until loading) and time interval 2 (from loading until examination) were individually computed for each patient. For final comparison, a dependent t-test for paired samples of the mean gradients in the different intervals was used.

To detect parameters potentially affecting the outcome, Welch’s *t*-tests on the data grouped by gender, implant region, implant location, implant material, difficulty of the operation and number of roots, were performed. Difficulty of the operation and number of roots were included in Welch’s t-tests, since only 2 different entries were made in the evaluation of the CRFs. The Pearson’s Correlation Coefficient between the total bone loss, age, length of the one-piece implant, root portion: Length, abutment portion: Length, implant surface, and bone quality were computed. Additionally, the Pearson’s Correlation was applied to investigate the coefficient among the total bone loss, esthetics (VAS), PES, WES, and PES/WES. Testing was adjusted to the quantity and quality of evaluable data (i.e., just 26 series of X-rays were evaluated). Survival and success rates were specified with calculation of percentages.

All statistical tests were performed with SciPy (SciPy developers), a Python-based ecosystem of open-source software for mathematics, science, and engineering. The level of significance was set at *p* < 0.05.

## 3. Results

### 3.1. Demographic Data and Additional Information

One hundred and seven RAI-surgeries were performed between September 2016 and August 2019 in the dental office of this study. Survival of implants was verified at follow-up examinations or, in case of a refused participation, by phone via the patients themselves or the records of the general dentists (see also Figure 1). A hundred and one root-analogues were still in situ at the time of the study after a mean observation period of 18.9 ± 2.4 months. Six RAIs failed, of which 4 did not show osseointegration in the healing period and 2 were lost after prosthetic delivery. This resulted in a survival rate of 94.4%.

A total of 28 patients, consisting of 11 males and 17 females, were recruited for follow-up examinations. One study participant was treated with two and another participant with three RAIs, resulting in a total number of 31 retrospectively examined and restored root-analogues (Table 2).

The mean age was 55.3 ± 14.2 years at surgeries and 56.6 ± 14.1 years at follow-up examinations. Implant placements of the study participants were performed between September 2016 and September 2018. Loading took place between August 2017 and August 2019. Twenty-five hybrid and 6 all-ceramics RAIs were restored with 29 single crowns and one three-unit fixed dental prostheses after a mean healing time of 6.6 ± 2.5 month. Follow-up examinations took place 17.5 ± 6.4 months after surgery and 10.8 ± 7.0 month after loading. Patient and RAI characteristics are shown in Table 3 and Table 4 including *p*-values related to the impact of the different parameters on MBL.

### 3.2. Implant Level

**Figure 4:**
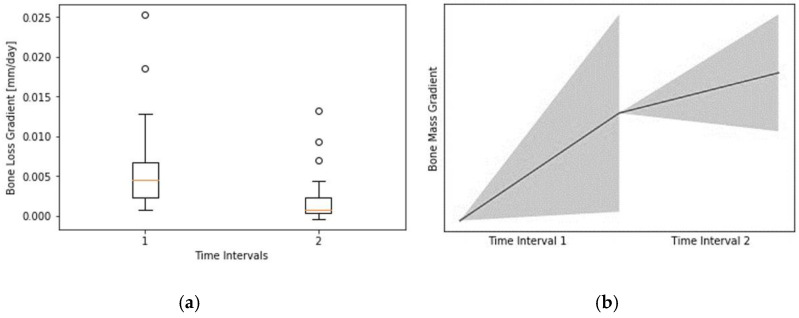
(**a**) Bone loss gradient in mm/day from surgery to loading (T0-T1; Time Interval (1) and loading to examination (T1-T2; Time Interval (2); (**b**) visualization of the box plot without any possible axis labeling based on the calculation of the gradients due to heterogeneous follow-up intervals. Created with SciPy (SciPy developers).

Of 31 RAIs, 26 were radiographically evaluated. Detailed information regarding the data used for measurements of the MBL are shown in Table 5. Before loading of the RAIs the mean MBL was calculated to be 0.82 ± 0.54 mm at T1 (T0-T1). At follow-up examinations the mean MBL was 1.20 ± 0.73 mm at T2 (T0-T2). Between loading (T1) and follow-up (T2), the MBL was 0.40 ± 0.41 mm (T1-T2). Bone resorption after surgery was recorded in every patient. Comparing T0-T1 with T1-T2 (Figure 4), a statistically significant reduced progression of bone resorption could be determined (*p* = 0.013).

Potential affecting parameters show no statistically significant influence on MBL and are shown in Table 3 and Table 4. The junction between the implant and abutment portions (JIAP) of the RAIs were planned iso- or subcrestally while designing the root-analogues prior to surgery (see also Table 5). Since the majority of JIAP were planned 1 mm subcrestally, a statistical evaluation of the influence of JIAP position on MBL was not reasonable. Adopting the afore explained criteria, 80.8% of the evaluable RAIs (*n* = 21) were successful regarding the implant level (Table 6). Pearson’s Correlation Coefficients are documented in Table 7.

### 3.3. Peri-Implant Soft Tissues

The level of presurgical planned restoration margins was ranging from 0.0 mm (*n* = 1), 1.0 mm (*n* = 13), 0.5–1.5 mm (*n* = 5) to ≥1.5 mm (*n* = 12) subgingival. All 31 root-analogues were evaluated regarding the peri-implant soft tissues at follow-up as shown in Table 8. The mean mPI was documented to be 0.6 ± 0.5, the mean mBI was 0.6 ± 0.7, and the mean KG was 3.9 ± 1.7 mm. This resulted in a success rate of 96.8% (*n* = 30) on the soft tissue level, as shown in Table 6.

### 3.4. Prosthetic Level

The total values of PES (mean 7.45 ± 1.50) and WES (mean 7.90 ± 1.74) resulted in a mean score of 15.35 ± 2.33 for PES/WES (Table 9). Including the criteria regarding the prosthetic level, a success rate of 83.3% (*n* = 25) was found (Table 6). Additionally, evaluation of the modified USPHS criteria for the analysis of single crowns and fixed dental prostheses was documented in detail in Table 10. Pearson’s Correlation Coefficients evaluating PES, WES, and PES/WES show no strong dependencies and can be found in Table 7.

### 3.5. Patient Satisfaction

The esthetic appearance of the reconstructions (*n* = 30) was rated 91.6 ± 17.5% on the VAS. A slightly lower mean value of 89.1 ± 18.9% was evaluated regarding the ability to chew. As previously defined, considering both parameters resulted in a success rate of 90.0% (*n* = 27; Table 6) regarding the patient satisfaction. Due to three participants, rating (1) The satisfaction with the appearance and/or (2) The ability to chew as unsatisfactory were counted as failure. Of those, two described discomfort while chewing and one was not pleased with the appearance of the single crown. Pearson’s Correlation Coefficients looking at esthetics (VAS) were again specified in Table 7.

### 3.6. Overall Survival and Success

Taking the predefined criteria in all 4 categories into account, a success rate of 64.5% (*n* = 20, based on 31 RAIs) and a survival rate of 94.4% (based on 107 RAIs) was determined 17.5 ± 6.4 months after RAI surgeries (Table 6).

## 4. Discussion

Immediate implantations with customized root-analogue implants show comparable results to conventional, screw-shaped implants after a short observation period. It seems that there are advantages in terms of soft tissues and esthetics, but disadvantages in terms of bone loss and restoration margins. However, the database is very small, and the calculations of success and survival rates should be interpreted with care. When discussing the present findings, the limitations of this study, especially regarding the limited sample size, should be kept in mind and conclusions mainly regarded as a tendency. A prospective study design should have been preferred. At the time this study was developed, available data needed to be evaluated retrospectively. However, due to the small indication group for treatment with a RAI and little data described in the literature, it was decided that the presented outcomes should be of interest to the field. Including this study there are too little studies available to be able to make a final comparison between RAIs and screw-shaped implants. Despite the fact that NDI Berlin ceased its business operations on 31 January 2020, the authors expect future developments and investigations regarding RAIs.

Studies on root-analogue implants and potential advantages appear repeatedly in the literature. As the procedure is not established in clinical routine, only case reports with different follow-up intervals [14,15,16,17,20,22] or literature reviews exploring the subject [33] are available. Recently, an article reviewing the historical development of RAIs was published [34]. The primary goal of this retrospective case series was to collect comprehensive data and give preliminary survival and success rates for rehabilitations supported by root-analogue implants. Therefore, regularly used scientific parameters, as described in a review in 2012 [26], were examined. Considering the extensive recruitment area and the referral structure of the dental office of the study, the largest possible patient group treated with the *REPLICATE Immediate Tooth Replacement System* was acquired for follow-ups. To the knowledge of the authors, such an extensive and detailed analysis of RAIs has not been described in the literature yet.

For screw-shaped implants, standardized manufacturer’s specifications (e.g., the pitch distance of the threads) are available for radiological analysis of bone loss. As RAIs are fully customized, there are no such standardized values. Therefore, the manufacturer was asked to give individual implant-specific details (e.g., implant length, abutment length, etc.) and precisely specified distances from distinctive RAI points (Figure 3b). This enabled the calculation of MBL as it is performed for screw-shaped implants [35,36]. A control group is missing due to the retrospective study design and high specificity of the treatment. The assessment of bone loss based on two-dimensional X-rays was applied in numerous precedent publications, but findings should be handled with care due to potential artefacts and dimensional limitations of the projection [37,38]. Because of the retrospective study design, no standardized radiographs with customized X-ray holders were available, and measurements should be interpreted with care.

The loss of bone was measured referring to the marginal bone level after installation of the RAIs (T0). Evaluating the radiographs, the absolute loss was measured, even if JIAP was located subcrestally and a bone loss was therefore already expected and considered. This corresponded to the transition between the polished and rough parts of the implant. Due to very homogeneous location of this transition zone (1 mm subcrestal in 73.1%), its influence on bone remodeling was not statistically evaluated. However, it can be assumed that the bone remodeling is more pronounced in these RAIs, as there are studies, in which screw-shaped implants with a subcrestal rough-smooth border show higher bone remodeling in the first 6 months after installation, than screw-shaped implants with an epicrestal (or close to the crest) rough-smooth border [39,40]. In reference to this, immediate implant placements with screw-shaped implants are usually also performed ~1 mm subcrestally [41], whereby MBL measurements are often carried out with regard to the rough-smooth border after completed healing and before loading. Using prosthetic delivery and loading of the implant as baseline, a mean of 0.40 ± 0.41 mm of bone was lost within a mean of 10.8 ± 7.0 month of service, ranging within the accepted amount of bone loss due to bone remodeling processes [42,43]. Applying the success criteria of the present study, this remodeling processes from installation of the RAIs to final loading were included and resulted in a success rate of 80.8% (*n* = 21) on the implant level. In this study, none of the patient-reported, surgical or RAI-specific parameters had a statistically significant influence on the MBL. However, regarding the limited sample size, the presented data should be interpreted with care. In conclusion, no advantages of RAIs compared to screw-shaped implants by means of bone level stability seem to be apparent. Bone loss observed appears to be comparable to conventional immediate implant surgery with screw-shaped implants and does not seem to further counteract the bony resorption or remodeling processes. These results were documented after a short observation period and should be interpreted as a tendency regarding the methodology. Nevertheless, as well with RAIs, greater bone loss seems to be expected during the healing phase compared to the phase after loading, as it is the case with screw-shaped implants.

To evaluate the success of implant-supported rehabilitations the assessment of the peri-implant soft tissues is inevitable. An evaluation of these, e.g., in form of bleeding on probing (BoP), in combination with peri-implant bone loss is necessary to rule out an undesirable complication of implants, peri-implantitis [1,44].

The RAIs included in the present investigation were mostly designed with subgingival cementation margins ranging from 0.5–1.5 mm (96.8%). But even if a subgingival restoration margin of more than 0.5 mm increases the risk of undetected cement [45], potentially resulting in gingival inflammation, this study revealed favorable results regarding peri-implant soft tissues. Only for one single RAI, a mBI > 2 has been documented. The buccal KG was >1.5 mm at all RAIs and despite oral hygiene was not always categorized optimal, the mPI never exceeded a rating of 2. In the short-term, it seems that the lower success rate regarding MBL detected at RAIs does not have a negative effect on the peri-implant soft tissue stability and health. This may be due to the transition of the abutment to the root portion (shape analogue and same width), compared to screw-shaped implants (strong, hardly cleanable, and probeable rejuvenation). In consequence, the fully anatomical imitation with RAIs might be considered advantageous regarding the susceptibility for peri-implantitis [46].

Finally, implants serve as support to the attempt to restore esthetics and function. To investigate the esthetics and possible complications, objective criteria were necessary to assess success at the prosthetic level. A feasible and comprehensive esthetic score for comparing results regarding rehabilitations with implants is constantly being discussed in the scientific community [47]. For an objective evaluation of the esthetic appearance, the PES/WES [30] as a reproducible instrument [48] and the modified USPHS criteria, which had already been used in other clinical studies to evaluate single crown restorations on implants [32], were used for classification. It should be considered that the PES/WES was originally developed for the evaluation of implant restorations in the esthetic area to be compared with contralateral natural teeth. In the present study, however, only 41.9% (*n* = 13) of the RAIs were in the esthetic area. Thirty-five and a half percent (*n* = 11) of the contralateral teeth were natural teeth, and 64.5% were restored. In such cases, results can be both false positive and false negative, therefore affecting the outcome. Due to the detailed sub-parameters within the PES/WES, however, it still provides a comprehensible and reproducible result. A mean of 15.35 ± 2.33 for PES/WES represents a favorable result compared to previous studies adopting these criteria [30,49]. According to the modified USPHS criteria, six out of seven criteria provided satisfactory results, predominantly being rated with no (A) or minor complications (B). However, marginal integrity was found to be noteworthy. Many of the restorations were documented to show a slightly soundable marginal gap and for some restorations the explorer even penetrated a significant crevice. Concerning marginal integrity, there seems to be a trend towards increased difficulty to provide conventionally copied RAIs with accurately fitting single crowns or fixed dental prostheses compared to other one-piece implants investigated in studies using the USPHS criteria [32]. Nevertheless, none of the restorations had to be replaced and, regarding the evaluation of the peri-implant soft tissues, does not seem to have a negative influence. A sufficient margin design is nevertheless desirable in order not to provide an exposed surface for possible complications, e.g., promotion of peri-implantitis. If a new fabrication of the restoration is indicated and the abutment portion of the respective RAIs has not been manipulated after the installation, a matching of the new impressions with the existing digital data sets of the abutment portion should be considered. Due to the difficulties described above, this might help to optimize the transition at the preparation margin.

Apart from the preferably objective evaluation of the procedure, each participant was asked to mark VAS for a subjective assessment. The individual sensation at rest or in function, i.e., when biting or chewing nutrition, was rated by the participants with an average of 89.1%. Furthermore, the personal esthetic perception was evaluated with an average of 91.6%. Two participants were not satisfied with “the ability to chew” and have described a different feeling compared to their natural teeth. No pain, mobility, or other clearly defined criteria for failure were evident. Twice, the appearance of the restorations in terms of color and translucency were rated below the defined threshold of 80%. This resulted in 3 unsatisfied patients, since one patient rated both parameters below the threshold for success. Two of these RAIs were located in the anterior region and 1 was located in the posterior region. They were evaluated with 12/12/13 points according to the PES/WES and received AAACBBA/CABCBAA/AAABAAA ratings according to the USPHS criteria. The below-average assessment of the PES/WES is thus consistent with the patient-specific assessment. An interpretation of the modified USPHS criteria can also be reconciled with it. This supports the assumption that the criteria used in this study represent a reproducible method for assessing esthetics and complications and are close to individual patient evaluations. Thus, the investigated success rate of 90.0% regarding patient satisfaction is sustained by the pre-discussed objective parameters. The esthetic deficits and marginal discrepancies can be corrected by making new restorations using new impressions. For this purpose, the existing digital data sets should be considered as mentioned above and an additional focus on fittings should be carried out before the final cementation.

Patient satisfaction, esthetics and durability of the prosthetic outcome, soft tissue stability and health, and susceptibility for bone resorptions were considered relevant parameters for the determination of implant success. In terms of MBL, no advantage was found in comparison to screw-shaped implants. The strengths of the procedure seem to be mainly in the esthetic result and maintenance of healthy peri-implant soft tissues. Combining the four applied criteria for success resulted in an overall success rate of 64.5%. This is within the range of success rates of other scientific articles that have classified the implant success based on a combination of 4 superordinate criteria [50,51,52]. It is well known and reported that overall success decreases with an increasing number of included criteria [26]. Most research is limited to an examination of the implant level and especially the MBL.

It seems that the present findings are in line with the observations by Esposito and collaborates, likewise reporting a reduced success rate but highly satisfying esthetic results for immediately installed implants [53,54]. In contrast to conventional procedures, installing screw-shaped implants, one potential benefit might be considered the circumstance that no further augmentation techniques, such as a connective tissue grafts or guided bone regeneration, were required to achieve a comparably and esthetically pleasing result. Nevertheless, prospective long-term data are necessary for a final evaluation of RAIs. Most importantly, the influence of JIAP location on marginal bone remodeling and a more reliable evaluation of the resorption processes (three-dimensional or standardized radiographs) would be of interest. In addition, the development of a standardized success score for implant-supported reconstructions would be helpful to compare the findings of different studies in a more comprehensive manner.

Finally, treatment with RAIs is only suitable for a small group of patients with extractions planned in the near future. The alveolar bone needs to be intact and no extensive osteolysis and/or inflammatory processes in the implant region can be present. If possible, artefact-causing reconstructions in CBCTs potentially resulting in inaccuracies when production of the RAIs, such as metal, should be removed in advance. In general, it requires a very cautious approach by an experienced surgeon and destruction of the surrounding tissues during extraction can make RAI installation impossible. In such a case, planning efforts prior to surgery were useless, and the about 15% higher costs compared to screw-shaped implants (including bone and soft tissue augmentations) cannot be justified.

## 5. Conclusions

Immediate installation of RAIs does not seem to counteract the known marginal bone resorption processes after tooth extraction when compared to immediate installation of screw-shaped implants reported in the literature. However, at least after a short-time observation period, RAIs were found to maintain peri-implant soft tissue conditions and resulted in a predictable and highly satisfying esthetic outcome. For a more reliable analysis, mid- to long-term prospective studies are required. Given the small indication group and the limited data and advantages, it seems that the application of RAIs bears no relation to necessary efforts and expenses so far. The calculated survival rate for RAIs (94.4%) within the collected follow-up data after less than two years should be viewed critically when comparing to a survival rate of 94.6% considering 10-year follow-up data of screw-shaped implants [1]. During the completion of this study, NDI Berlin ceased its business on 31 January 2020. This and the failure to establish RAIs in dentistry within several years can be an indicator for its not yet convincing results.

## Figures and Tables

**Table 1:** Modified United States Public Health Service (USPHS) criteria for the analysis of single crowns and fixed dental prostheses [31,32].

	Alpha (A)	Bravo (B)	Charlie (C)	Delta (D)
**Fracture of veneering ceramic**	No fracture	Minor chipping (polishable)	Major chipping (up to framework)	Fracture (loss of reconstruction)
**Fracture of framework**	No fracture	-	-	Fracture (loss of reconstruction)
**Occlusal roughness**	No roughness	Slight roughness (Ø < 2 mm)	Obvious roughness (Ø > 2 mm)	Reconstruction needs to be replaced
**Marginal integrity**	No visible or soundable gap	Marginal gap slightly soundable	Explorer penetrates a significant crevice	Reconstruction needs to be replaced
**Contour of reconstruction**	Perfectly contoured	Slightly under-/overcountoured	Pronounced under-/overcontoured	Reconstruction inacceptable
**Esthetics of reconstruction**	Good esthetics	Slight mismatch in color	Severe color mismatch	Reconstruction inacceptable
**Discoloration of reconstruction**	No discolorations	discoloration		

**Table 2:** RAI distribution and region.

	Posterior (P)				Anterior (A)						Posterior (P)			
FDI	17	16	15	14	13	12	11	21	22	23	24	25	26	27
*n* (maxilla)	1	1	3	0	0	1	6	5	0	1	3	1	0	0
*n* (mandible)	0	2	2	1	0	0	0	0	0	0	1	3	0	0
FDI	47	46	45	44	43	42	41	31	32	33	34	35	36	37
*n* (region)	10				13						8			

FDI: Scheme according to the World Dental Federation; *n*: number of placed root-analogues in the respective position or region (anterior or posterior).

**Table 3:** Patient characteristics and *p*-values for investigated influence on MBL.

Parameter	Frequency *n* (%)	*p*-Value
Gender		0.260
female	17 (61)	
male	11 (39)	
Age		0.869
mean (in years)	55.3/56.6 ^1^	
range (in years)	31–82/33–83 ^1^	
Implant region		0.571
anterior	13 (42)	
posterior	18 (58)	
Implant location		0.691
maxilla	22 (71)	
mandible	9 (29)	
Implant material		0.483
hybrid	25 (81)	
all-ceramics	6 (19)	
Bone quality		0.898
I	1 (3)	
II	10 (32)	
III	14 (45)	
IV	6 (19)	
Difficulty of the operation		0.690
easy	0 (0)	
intermediate	21 (68)	
complicated	10 (32)	
Number of roots		0.091
single rooted	25 (81)	
multi rooted	6 (19)	

^1^ first data concerning the date of surgery, second data concerning the date of examination.

**Table 4:** RAI characteristics and *p*-values for investigated influence on MBL.

	Length of the One-Piece Implant (in mm)	Root Portion: Length (in mm)	Abutment Portion: Length (in mm)	Implant Surface (in cm^2^)
*n*	31	31	31	25 ^1^
Min	12.41	7.49	3.18	101
Max	26.30	14.79	14.86	442
SD	3.12	1.86	2.41	83
Mean	19.19	10.96	8.43	213
*p*-values	0.709	0.870	0.717	0.078

*n*: number of root-analogues included in calculations; ^1^ the implant surfaces of 6 all-ceramic RAIs could not be provided by NDI Berlin.

**Table 5:** Marginal bone loss and time difference from surgery (T0) to loading (T1) and the follow-up examination (T2).

RAIs	Surgery (T0) (in mm)	Loading (T1) (in mm)	Examination (T2) (in mm)	T0-T1 (in Days)	T1-T2 (in Days)	T0-T2 (in Days)	JIAP (in mm)
1	0.00	−0.50	−1.30	276	350	626	−1.00
2	0.00	−1.35	−1.55	276	350	626	−1.00
3	0.00	−0.20	−0.50	260	350	610	−1.00
4	0.00	−1.10	−1.30	281	272	553	0.00
5	0.00	−0.90	−0.90	155	418	573	−1.50
6	0.00	−1.10	−2.20	86	509	595	−1.00
7	0.00	−0.50	−0.75	86	663	749	−1.00
8	0.00	−1.10	−1.25	153	750	903	−1.00
9	0.00	−0.30	−0.45	126	34	160	−1.00
10	0.00	−0.75	−1.00	254	358	612	−1.00
11	0.00	−1.70	−1.70	343	260	603	0.00
12	0.00	−0.60	−0.85	291	369	660	−1.00
13	0.00	−1.70	−2.85	92	488	580	−1.00
14	0.00	−0.75	−1.40	132	182	314	−1.00
15	0.00	−0.95	−2.55	266	503	769	−0.75
16	0.00	−0.55	−0.85	228	32	260	−1.00
17	0.00	−0.40	−0.55	151	107	258	−1.00
18	0.00	−0.30	−0.75	135	34	169	−1.00
19	0.00	−0.40	−0.60	182	338	520	−1.00
20	0.00	−0.80	−1.15	124	50	174	−1.00
21	0.00	−0.35	−0.50	97	459	556	−1.00
22	0.00	−2.50	−2.90	99	598	697	−1.25
23	0.00	−0.45	−1.40	99	598	697	−1.25
24	0.00	−0.80	−0.80	102	584	686	−1.00
25	0.00	−0.65	−1.10	91	636	727	0.00
26	0.00	−0.15	−0.15	141	288	429	−1.00
	Min	−0.15	−0.15	86.00	32.00	160.00	−1.5
	Max	−2.50	−2.90	343.00	750.00	903.00	0.00
	SD	0.54	0.73	78.91	208.77	202.03	0.36
	Mean	−0.82	−1.20	173.56	368.46	542.54	−0.91

JIAP: the prior to surgery planned iso- or subcrestal junction between the implant and abutment portion of the RAIs; only 26 of 31 RAIs showed a retrospectively evaluable series of X-rays at follow-up; negative values indicate a loss of bone in relation to marginal bone levels at surgery.

**Table 6:** Success criteria based on included parameters.

	Implant Level		Soft tissue Parameters		Prosthetic Level		Patient Satisfaction		Overall Rating	
	***n***	**%**	***n***	***%***	***n***	**%**	***n***	**%**	***n***	**%**
*n*	26	83.9	31	100.0	30	100.0	30	100.0	31	100.0
Success	21	80.8	30	96.8	25	83.3	27	90.0	20	64.5

*n*: number of evaluated RAIs, respectively, reconstructions; only 26 RAIs could be included in the evaluation of MBL; 31 RAIs were investigated regarding soft tissue parameters; due to 29 single crowns and 1 fixed dental prosthesis, the number of investigated reconstructions and patient satisfaction was 30.

**Table 7:** Pearson’s Correlation Coefficients.

	Esthetic (VAS)	PES	PES/WES	WES	Total Bone Loss (T0-T2)
Esthetic (VAS)	1.000000	0.335767	0.358691	0.164237	0.074137
PES	0.335767	1.000000	0.666582	0.137687	−0.089390
PES/WES	0.358691	0.666582	1.000000	0.685310	0.012887
WES	0.164237	0.137687	0.685310	1.000000	0.096554
Total bone loss (T0-T2)	0.074137	−0.089390	0.012887	0.096554	1.000000

**Table 8:** Peri-implant soft tissue parameters.

	mPI	mBI	KG (in mm)
*n*	31	31	31
Min	0	0	1.5
Max	1	3	9
SD	0.5	0.7	1.7
Mean	0.6	0.6	3.9

*n*: number of included root-analogues in calculations; mPI: the modified plaque index [29] mBI: the modified bleeding index [29]; KG: width of buccal keratinized gingiva.

**Table 9:** Detailed Pink Esthetic Score (PES) and White Esthetic Score (WES) for each RAI investigated [30].

PES							WES						
RAIs	Mesial Papilla	Distal Papilla	Curvature of Facial Mucosa	Level of Facial Mucosa	Root Convexity/Soft Tissue Color and Texture	Total PES	Tooth Form	Outline/Volume	Color (Hue/Value)	Surface Texture	Translucency/Characterization	Total WES	Total PES + WES
1	1	2	2	1	2	8	2	2	2	2	2	10	18
2	1	2	2	1	2	8	2	2	2	2	2	10	18
3	2	2	1	1	1	7	1	1	2	2	2	8	15
4	2	2	2	2	2	10	2	2	2	1	2	9	19
5	1	2	1	2	1	7	1	1	1	1	1	5	12
6	2	2	2	1	1	8	1	1	1	1	1	5	13
7	2	2	2	1	1	8	1	1	1	2	1	6	14
8	0	0	2	2	1	5	1	1	2	2	1	7	12
9	1	1	2	2	2	8	2	2	1	1	2	8	16
10	2	1	2	2	2	9	2	2	1	1	1	7	16
11	2	1	2	2	2	9	2	2	1	1	1	7	16
12	2	2	1	1	1	7	1	1	2	1	1	6	13
13	2	2	1	2	2	9	1	1	1	1	2	6	15
14	1	2	1	1	1	6	1	1	2	2	2	8	14
15	1	1	1	1	1	5	1	1	1	2	2	7	12
16	1	0	2	1	2	6	2	2	2	2	2	10	16
17	2	1	2	2	2	9	2	2	1	2	1	8	17
18	0	2	2	1	2	7	2	1	1	1	2	7	14
19	2	0	1	1	1	5	1	1	1	1	1	5	10
20	1	1	2	2	2	8	2	2	2	2	2	10	18
21	1	0	2	2	2	7	2	2	2	2	2	10	17
22	1	1	1	1	2	6	2	2	2	2	2	10	16
23	1	1	1	1	2	6	2	2	2	2	2	10	16
24	1	1	2	2	1	7	1	1	2	2	2	8	15
25	1	2	2	2	2	9	2	2	2	2	2	10	19
26	0	0	1	1	2	4	2	2	1	2	2	9	13
27	2	1	2	2	1	8	1	1	2	1	1	6	14
28	2	2	2	2	1	9	2	2	2	2	2	10	19
29	1	2	2	2	1	8	2	2	1	2	1	8	16
30	2	2	1	2	2	9	1	1	1	2	1	6	15
31	2	2	2	2	1	9	2	2	1	2	2	9	18
Min	0	0	1	1	1	4	1	1	1	1	1	5	10
Max	2	2	2	2	2	10	2	2	2	2	2	10	19
SD	0.66	0.75	0.49	0.51	0.51	1.50	0.50	0.51	0.51	0.49	0.50	1.74	2.33
Mean	1.35	1.35	1.65	1.55	1.55	7.45	1.58	1.55	1.52	1.65	1.61	7.90	15.35

**Table 10:** Results applying modified USPHS criteria as mentioned in Table 1 at follow-up.

	Alpha (A)	Bravo (B)	Charlie (C)	Delta (D)
**Fracture of veneering ceramic**	29 (96.7%)	0	1 (3.3%)	0
**Fracture of framework**	30 (100.0%)	0	0	0
**Occlusal roughness**	26 (86.7%)	4 (13.3%)	0	0
**Marginal integrity**	9 (30.0%)	17 (56.7%)	4 (13.3%)	0
**Contour of reconstruction**	22 (73.3%)	8 (26.7%)	0	0
**Esthetics of reconstruction**	24 (80.0%)	6 (20.0%)	0	0
**Discoloration of reconstruction**	30 (100.0%)	0		

30 included restorations due to 29 single crowns and 1 three-unit fixed dental prostheses.
